# A labeled dataset for building HVAC systems operating in faulted and fault-free states

**DOI:** 10.1038/s41597-023-02197-w

**Published:** 2023-06-01

**Authors:** Jessica Granderson, Guanjing Lin, Yimin Chen, Armando Casillas, Jin Wen, Zhelun Chen, Piljae Im, Sen Huang, Jiazhen Ling

**Affiliations:** 1 Lawrence Berkeley National Laboratory, Berkeley, USA; 2 Drexel University, Philadelphia, USA; 3 Oak Ridge National Laboratory, Oak Ridge, USA; 4 National Renewable Energy Laboratory, Golden, USA

**Keywords:** Research data, Energy efficiency

## Abstract

Open data is fueling innovation across many fields. In the domain of building science, datasets that can be used to inform the development of operational applications - for example new control algorithms and performance analysis methods - are extremely difficult to come by. This article summarizes the development and content of the largest known public dataset of building system operations in faulted and fault free states. It covers the most common HVAC systems and configurations in commercial buildings, across a range of climates, fault types, and fault severities. The time series points that are contained in the dataset include measurements that are commonly encountered in existing buildings as well as some that are less typical. Simulation tools, experimental test facilities, and *in-situ* field operation were used to generate the data. To inform more data-hungry algorithms, most of the simulated data cover a year of operation for each fault-severity combination. The data set is a significant expansion of that first published by the lead authors in 2020.

## Background & Summary

Fault detection and diagnostics (FDD) is a well-established field of study in building science and building technology applications. This is largely driven by the significant impact of equipment faults and control problems on building energy use and emissions, equipment life, and occupant comfort. Building HVAC systems in particular, afford a rich opportunity space for FDD algorithm development, given the multiplicity of system configurations, complex operations, and availability of monitored data. In addition, the recent push to decarbonize buildings and the electricity sector is increasing the importance of grid-interactive efficient buildings that can reliably provide load-flexibility services to the renewable supplied grid. This is making it even more critical to ensure that building HVAC systems are controllable and fault-free, providing further motivation for FDD technology development and deployment.

In buildings, FDD software tools employ operational data collected from building automation systems, sensors, and meters, to automatically detect equipment and control problems, or degrading performance in an HVAC system, and to diagnose potential root causes^1^. Using the results from FDD technologies, building operators can efficiently direct maintenance activities to address inefficiencies, or equipment and control malfunctions.

In the past thirty years, a large body of literature has been published documenting the development and application of FDD solutions for buildings. The active research covers a breadth of topics including: (1) the development and validation of hundreds of FDD methods^2–4^; (2) the development of experimental platforms or simulation software tools to generate fault inclusive models^5–7^, and the development of fault-inclusive data sets^8–10^; (3) quantification of the prevalence and occurrence rates of faults in buildings^11–13^; (4) analysis of the impact of faults on system operations^14,15^, energy consumption^16,17^, equipment maintenance and operational costs^18,19^, occupant thermal comfort^15,20,21^, and indoor air quality^22^; (5) FDD technology application, costs, and benefits, in existing buildings^1,23^; (6) FDD algorithm performance testing methodologies^24,25^; and (7) automated fault correction^26,27^ and maintenance activities^28^ after faults are diagnosed and flagged by FDD tools.

Although building control and automation systems are able to store and export large volumes of operational data, these data are often prone to data quality issues including erroneous sensors and gaps. Consistent naming conventions are not used from one system to another, and semantic metadata to interpret the meaning and relationships between data are rarely used. A further complication is that the data reflect unknown and unlabeled presence of a wide variety of commonly occurring faults. Finally, while small collections of field data may be acquired by researchers, it is extremely difficult to amass a large-scale dataset that represents climate, HVAC system, and operational diversity. This presents tremendous barriers for innovation in FDD algorithm development, and performance evaluation.

Extending the body of work focused on FDD algorithm testing methods and test datasets, this paper documents a significant expansion of the HVAC fault dataset presented in^9^. The expansion incorporates five new HVAC systems and configurations, an increased number of fault cases, and more extensive time spans for each fault-intensity combination, (in most cases reaching a full 365 days). The data were produced using simulation tools, laboratory experimental facilities, and field tests. Additionally, a semantic model for each system has been developed according to the Brick schema^29^ for improved usability and conformance with today’s commonly used building industry metadata schema.

The expanded dataset documented in this article includes seven common HVAC systems: the single duct air handling unit (AHU) system, the packaged rooftop unit (RTU), the dual duct AHU system, the fan coil unit (FCU) system, the variable air volume fan power unit (FPU), the boiler plant, and the chiller plant. 257 fault cases are represented, spanning sensor-related faults, actuator-related faults, control faults (e.g., controller PID parameter settings), and component faults (e.g., cooling coil foiling fault). In total, that dataset comprises 8 billion data samples, and represents the largest known ground truth-verified data for HVAC faults. As noted in the 2020 publication^9^ FDD researchers and developers can use the data to:

- Develop, evaluate, and compare performance across FDD algorithms;
- Identify performance gaps to focus future development efforts and resource investment;
- Develop an understanding of how FDD technology overall is improving over time; and
- Enable a better understanding of HVAC system performance under faulted and fault-free operation conditions for educational purposes.

Prior work such as ASHRAE research projects RP-1312 and RP-1043, and National Institute of Standards and Technology (NIST) 10D243 project, represent early contributions of operational HVAC fault data. This research advances those early efforts by increasing the number and type of HVAC systems that are represented, by increasing the duration of fault-free and faulted operational span (one year in most cases), and by increasing the number and type of faults that are represented. This will significantly increase the usability of the dataset for FDD algorithm development and performance evaluation.

## Methods

The newly expanded dataset contains experimental and simulated data across the seven HVAC systems types and configurations that are represented - the majority being simulated. Diverse facilities and simulation tools were used to create the data, and methods to impose the faults were created for each fault, given the specific HVAC system of focus, the control sequences that defined its operation. These facilities and tools, HVAC system details, and fault methods are described in the following, as is the metadata schema that was applied to the data. Provision of the metadata enables ease of interpretation of the data, and supports users of the dataset who wish to employ more automated procedures to interface FDD algorithm instances with the data.

### Facilities and simulation tools

The simulated datasets were created using HVACSIM+ and an EnergyPlus-Modelica co-simulation. HVACSIM + was developed by the US NIST^30^, the Modelica Buildings Library^31^ is developed by the Lawrence Berkeley National Laboratory, and EnergyPlus^32^ is developed by several contributors through funding from the US Department of Energy. Described with respect to other modeling tools in^33^, HVACSIM+, Modelica, and EnergyPlus are non-proprietary tools to model the behavior of building HVAC systems using physics-based approaches. In addition, Modelon’s air conditioning library was used to model the refrigerant side faults in the RTU system^34^. This library provides ready-to-use refrigeration cycle templates and a wide range of components to create a variety of air conditioning system configurations.

Four experimental research facilities were used to create data and to develop and validate simulation models:

1. FLEXLAB located at the Lawrence Berkeley National Laboratory in Berkeley, California, for the generation of the single-zone CAV data set and the variable-air-volume (VAV) AHU data set^9^.
2. The Flexible Research Platform (FRP) located at the Oak Ridge National Laboratory in Oak Ridge Tennessee, for the generation of RTU data sets^9^.
3. The Energy Resource Station facility was previously located at the Iowa Energy Center in Ames City, Iowa, for the development and validation of DD-AHU, FCU and FPU simulation models, and for creation of multi-zone VAV AHU data^35^.
4. The RTU facility is located in the Thermal Technology Facility (TTF) at the National Renewable Energy Laboratory in Golden, Colorado, for the validation of the RTU simulation model. NREL’s TTL is a flexible multipurpose laboratory that enables detailed evaluation and development of building and thermal energy systems. The TTF research space reaches 11,000 sq.ft. Two RTUs—a 5-ton/SEER 17 (RTU 1) and a 6-ton/IEER 23 (RTU 2) are installed in the TTL to develop comprehensive performance maps suitable for use with whole-building energy simulation computer programs. The SEER 17 contained a two-stage scroll compressor with R-410A, single-speed condenser fan, direct-drive variable-supply air fan with a high-efficiency motor, low leak dampers, hot gas reheat humidity control, and an economizer. The IEER 23 contained a variable-speed direct-drive compressor, variable-speed fans, and control logic that maintained the compressor and thermal expansion valve (TXV) within their performance limitations^36^.

Field data representing faulted and un-faulted rooftop unit operation is also included in the dataset. This data was collected from two RTUs, one in a restaurant building in Milford, CT and another one in a distribution center building in Colchester, CT. Table 1 summarizes these sites and the RTUs.

**Table 1:** Summary of field sites and RTU characteristics.

Site #	Building			RTU	
	Location	Space Type	Floor Area (ft^2^)	Capacity (Tons)	EER
1	Milford, CT	Restaurant	5,168	7.5	9
2	Colchester, CT	Distribution Center	207,635	10	10.4

### System configurations and control sequences

The configurations and sequences for each system in the data set are comprehensively documented for users of the data in an inventory file. This information is often needed to specify controls-specific parameters in fault detection and diagnostic algorithms. To illustrate the form and content of this information, two examples are presented - the fan coil unit system, and the boiler plant.

#### Fan coil unit

**Fig. 1:**
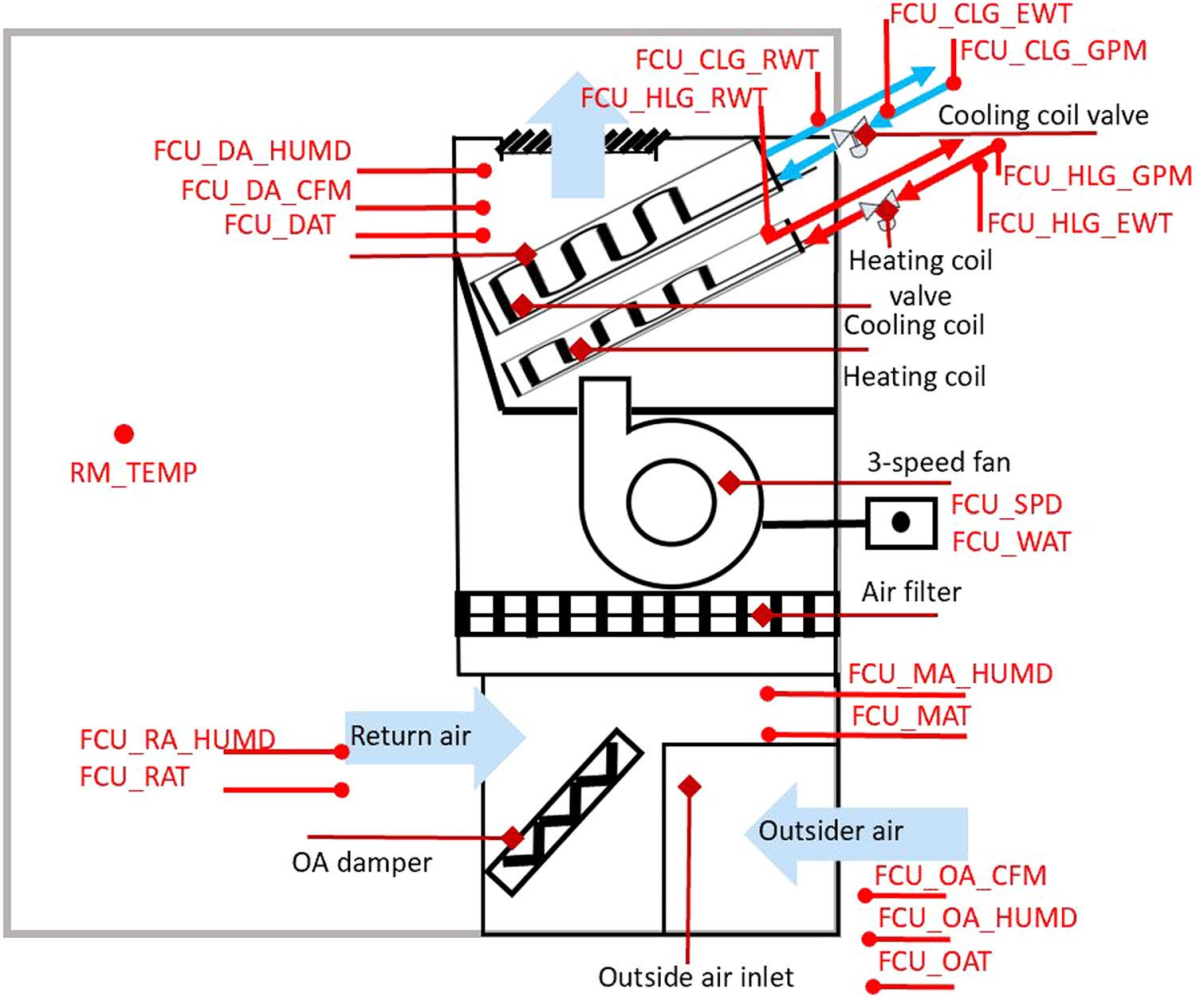
Schematic diagram of the FCU.

Figure 1 contains the schematic representation of the fan coil unit (FCU) system.

The FCU is scheduled for automatic operation on a time of day basis for occupied and unoccupied mode.

Occupied mode (Monday – Friday 6:00AM–17:59PM)

During these hours, the system is in Operate Mode. Five control sequences - control, outdoor air damper control, cooling coil valve control, heating coil valve control sequence, and zone temperature setpoints - were set during the simulation.

- Fan control
  - 3-speed fan with “Automatic On/Off” (Auto) mode: the fan on/off and speed change is based on the cooling proportional-integral-derivative (PID) output and heating PID output. The 10% dead band is given at each speed switchover level.
    - Low speed condition: the PID outputs (the cooling/heating coil valve position) are higher than 0% and lower than 40%;
    - Medium speed condition: the PID outputs (the cooling/heating coil valve position) are > = 40% and <80%;
    - High speed condition: the PID outputs (the cooling/heating coil valve position) are > = 80% and < 100%;
    - Off: no heating or cooling demand.
- OA damper control
  - The OA damper maintains a minimum damper position at 30%.
- Cooling coil valve control sequence
  - The PID control is used to adjust the cooling coil valve position. The setpoint dead band is 1 °F. If the actual room temperature is beyond 1 °F of the cooling setpoint, the FCU is in the “cooling” mode, and the cooling coil valve PID loop is enabled and the cooling valve position will be controlled by the cooling coil valve controller PID output. When the room temperature falls below 1 °F compared to the cooling setpoint, the cooling PID is disabled and the valve fully closed.
- Heating coil valve control sequence
  - The PID control is used to adjust the heating coil valve position. The setpoint dead band is 1 °F. If the actual room temperature is beyond 1 °F of the heating setpoint, the FCU is in the “heating” mode, and the heating coil valve PID loop is enabled and the heating valve position will be controlled by the heating coil valve controller PID output. When the room temperature falls below 1 °F compared to the heating setpoint, the heating PID is disabled and the valve fully closed.
- Zone temperature setpoints
  - Zone cooling setpoint: 72 °F;
  - Zone heating setpoint: 68 °F.
- Shutdown mode
  - The shutdown mode is only triggered by the low temperature protection described below. Under the shutdown mode, the fan is constantly off, and the OA damper is fully closed.
- Low Temperature Protection
  - During the simulation, when the mixed air temperature is below 35 °F and persists for 300 seconds, the FCU system will switch to the shutdown mode to prevent freezing the coil. The shutdown mode will last until the end of the day. The system will be turned back to normal operation at the beginning of the next day.

Unoccupied mode

During these hours, the system is in Setback Mode. The operation is similar to the operation mode except two additional settings as:

- Outdoor air damper: The OA damper is fully closed
- Zone temperature setpoints
  - Zone cooling setpoint: 85 °F;
  - Zone heating setpoint: 55 °F.

#### Boiler plant

**Fig. 2:**
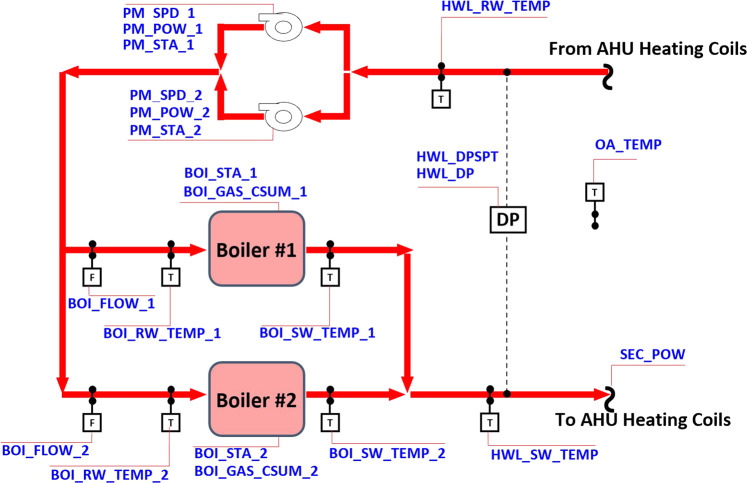
Schematic of the studied boiler plant system.

Figure 2 illustrates the configuration of the boiler plant system. This system has two identical boilers and two hot water pumps and provides hot water to heating coils in the air-side system.

**Fig. 3:**
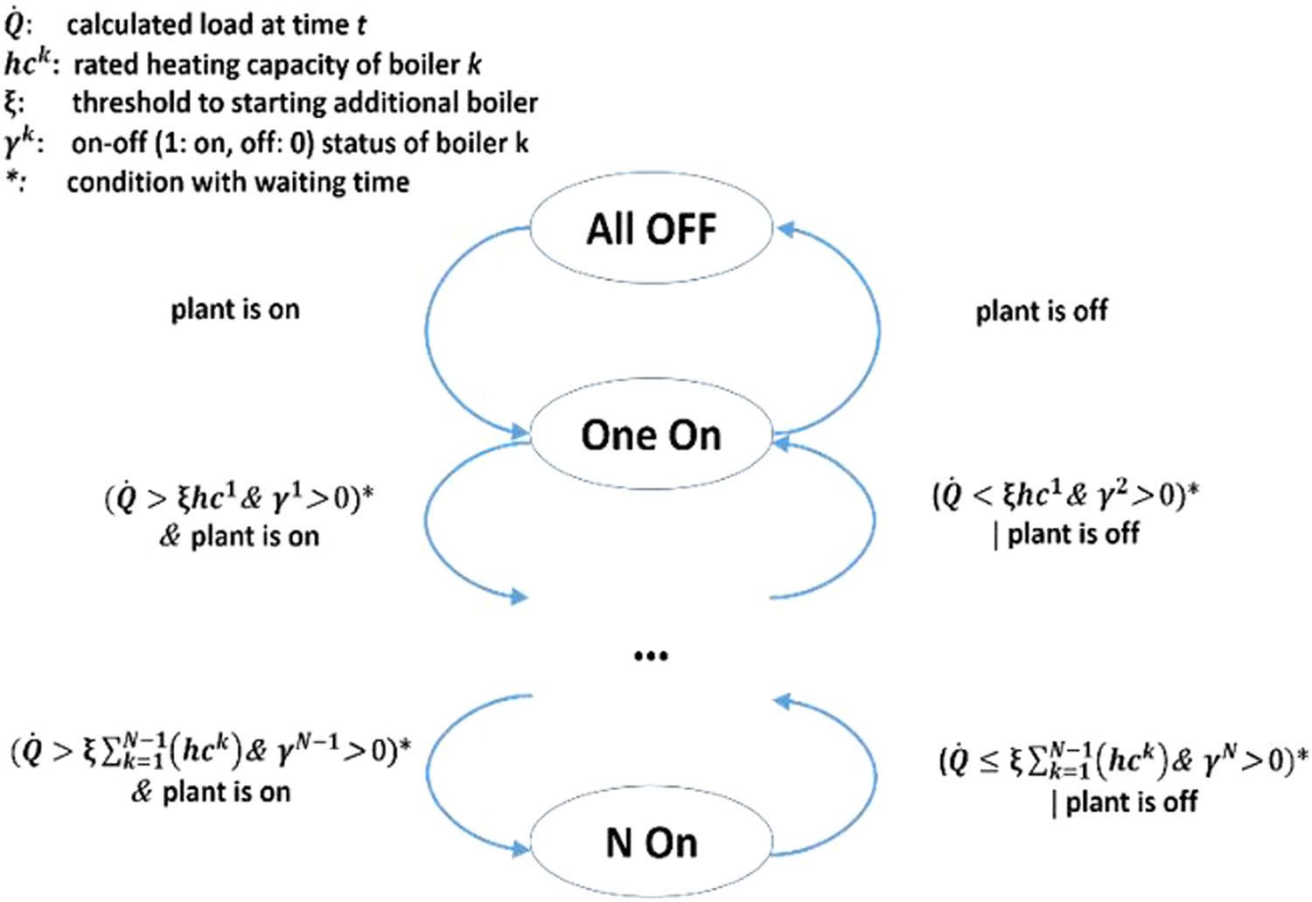
Staging control of boilers (*ξ* = 0.95 and waiting time: 30 min).

**Fig. 4:**
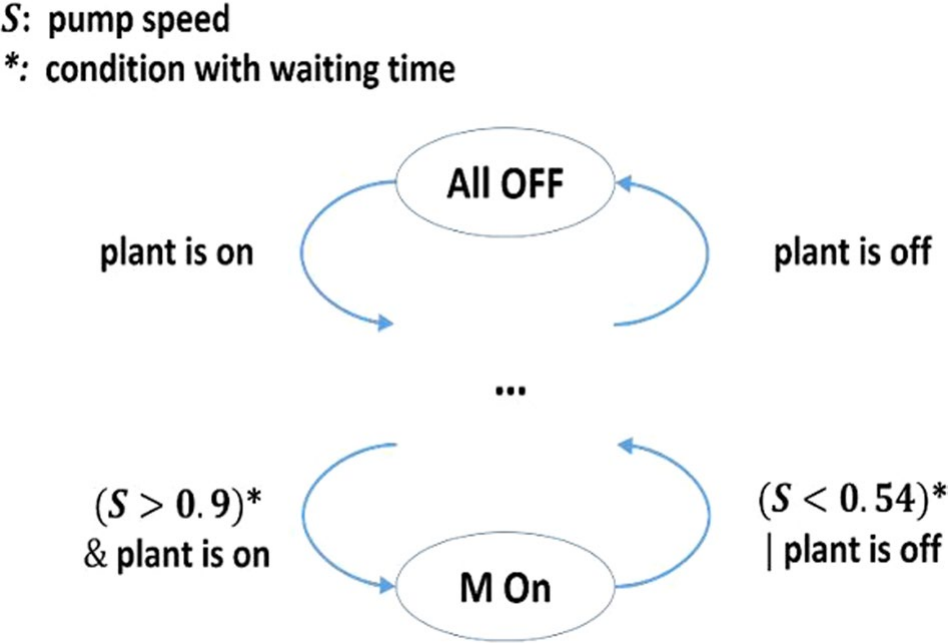
Staging control of hot water pumps in the boiler plant system (waiting time: 30 min).

The boiler plant system is controlled by two supervisory controllers and two local controllers (Table 2). One supervisory controller determines the number of the operating boilers using a state machine and the calculated heat load, as shown in Fig. 3. The heating load is calculated from:

1 $$\mathop{Q}\limits^{^\circ }={\mathop{v}\limits^{^\circ }}_{hw}\rho {C}_{p}\left({T}_{hw}^{ent}-{T}_{hw}^{lea}\right),$$

where ${\mathop{v}\limits^{^\circ }}_{hw}$ is the volumetric flow rate of the hot water, ${T}_{hw}^{ent}$ and ${T}_{hw}^{lea}$ are the temperature of the hot water entering and leaving the boiler plant system, respectively. The other supervisory controller determines the number of operating hot water pumps, as shown in Fig. 4.

**Table 2:** Local controllers in the boiler plant system.

No.	Controlled Variables	Description
1	Heating power of operating boilers	The heating power of each operating boiler is controlled by a feedback loop to maintain the temperature of the water leaving each boiler to be a predefined value (176°F).
2	Speeds of operating hot water pumps	Hot water pump speed is controlled by a feedback loop to maintain the pressure difference in the hot water loop to be 17.5 psi. If two hot water pumps are running, both pumps operate at the same speed.

### Fault scenarios and methods of fault imposition

Tables 3–10 summarize fault profiles and how each fault was imposed for each of the systems and fault scenarios. For the simulated datasets, each fault type and intensity were imposed for a full calendar year of operation - the exception being the simulated RTU dataset that covered a 100-day cooling season. For the experimental and field test datasets, fault type-intensity combinations were captured for one to 183 days of operation.

**Table 3:** Methods of fault imposition for the SD-AHU dataset.

Input Scenarios		Method of Fault Imposition
Fault Type	Fault Intensity	
Outdoor air temperature sensor bias	2 °C, 4 °C, −2 °C, and −4 °C	Add a bias value to the sensor output
OA damper stuck	Minimum position 10%, 25%, 75% and 100%*	Override the OA damper position to indicate that the OA damper is stuck
Cooling coil valve leaking	10%, 25%, 40%, and 50%	Adjusted the coil valve position value when the control signal is zero
Cooling coil valve stuck	10%, 25%, 50%, and 75%	Override of the coil valve position to indicate that the valve is stuck

*For the OA damper stuck at 100% open fault, the data is available from April 1 to Nov 1.

**Table 4:** Methods of fault imposition for the DD-AHU dataset.

Input Scenarios		Method of Fault Imposition
Fault Type	Fault Intensity	
Heating coil fouling water-side	Severe, moderate and minor	Increase the water flow pressure resistance by 50%, 30% and 10%; and decrease the heat transfer rate by 50%, 30% and 10%, for the severe, moderate and minor levels, respectively
Heating coil fouling air-side		Increase airflow resistance by 200%, 50% and 10%; and decrease the heat transfer rate by 10%, 5% and 0% for the severe, moderate and minor levels, respectively
Cooling coil fouling water-side		Increase water flow pressure resistance such that the water flow rate decreases by 50%, 30% and 10% when the valve is fully open; and decrease heat transfer rate by 50%, 30% and 10% for the severe, moderate and minor levels, respectively
Cooling coil fouling air-side		Increase the airflow resistance by 200%, 50% and 10%; decrease heat transfer rate by 10%, 5% and 0% for the severe, moderate and minor levels, respectively
Cold deck supply air temperature sensor bias	−4 °C, −2 °C, +2 °C, and +4 °C	Add a bias value to the sensor output
Hot deck supply air temperature sensor bias		
Cold deck supply air pressure sensor bias	−0.4 inH_2_O, −0.2 inH_2_O, +0.2 inH_2_O, and +0.4 inH_2_O	
Hot deck supply air pressure sensor bias		
Cooling damper stuck	0%, 20%, 50%, 80% and 100%	Assign a fixed device position
Heating damper stuck		
Cooling coil valve stuck		
Heating coil valve stuck		
Outdoor air damper stuck		
Heating sequence unstable control	NA	Increase the proportional band until unstable (from −45.7 K to −4K)
Cooling sequence unstable control

**Table 5:** Methods of fault imposition for the FCU dataset.

Input Scenarios		Method of Fault Imposition
Fault Type	Fault Intensity	
Heating coil fouling air-side	Severe, moderate and minor	Increase airflow resistance by 200%, 50% and 10%; and decrease heat transfer rate by 10%, 5% and 0% for the severe, moderate and minor levels, respectively
Heating coil fouling water-side		Increase water flow pressure resistance such that the water flow rate decreases by 50%, 30% and 10% when valve is fully open; decrease heat transfer rate by 50%, 30% and 10% for the severe, moderate and minor levels, respectively
Cooling coil fouling air-side		Increase airflow resistance by 200%, 50% and 10%; and decrease heat transfer rate by 10%, 5% and 0% for the severe; moderate; and minor levels, respectively
Cooling coil fouling water-side		Increase water flow pressure resistance such that the water flow rate decreases by 50%, 30% and 10% when valve is fully open; and decrease heat transfer rate by 50%, 30% and 10% for the severe; moderate; and minor levels, respectively
Filter restriction		Increase air flow pressure resistance by 10%, 20% and 50%; and increase the outlet resistance by 23.45%, 56.25% and 400% for the severe; moderate; and minor levels, respectively
Outdoor air inlet blockage	Block 80% area	Decrease the damper face area
Outdoor air damper leaking	Leaking at +20%, +50%, and +80%	Increase the damper face area
Zone air temperature sensor bias	−4 °C, −2 °C, +2 °C, and +4 °C	Add a bias to the sensor output
Heating coil valve stuck	0%, 20%, 50%, 80%, and 100%	Assign a fixed simulated controlled device position
Cooling coil valve stuck		
Outdoor air damper stuck		
Heating coil valve leaking	20%, 50%, 80% of the max flow (0.066 kg/s heating, 0.036 kg/s cooling)	Assign a water flow rate when the valve fully closed
Cooling coil valve leaking		
FCU unstable control	NA	Decrease both heating and cooling proportional bands to 10% of normal value, respectively
Heating control reverse acting		Modify simulated control strategy to allow reversed action
Cooling control reverse acting		
Fan outlet blockage	A 80% flow rate reduction at the same pressure difference (corresponding to outlet resistance by 2400%)	Increase air flow pressure resistance by 2400%

**Table 6:** Methods of fault imposition for the VAV fan power unit dataset.

Input Scenarios		Method of Fault Imposition
Fault Type	Fault Intensity	
Room temperature sensor bias	−4 °C, −2 °C, +2 °C, and +4 °C	Add a bias to the sensor output
VAV airflow sensor bias	−400CFM, −200CFM, +200CFM, and +400CFM	
VAV box fan restricted flow	FPU resistance is 1000% of the normal value	Increase plenum resistance (for PFPU) or VAV outlet resistance (for SFPU)
Hydronic reheat coil fouling water-side	Severe, moderate and minor	Increase water flow pressure resistance such that the water flow rate decreases by 50%, 30% and 10% when valve is fully open; and decrease heat transfer rate by 50%, 30% and 10% for the severe, moderate and minor levels, respectively
Hydronic reheat coil fouling air-side		Increase airflow resistance by 200%, 50% and 10%; and decrease heat transfer rate by 10%, 5% and 0% for the severe, moderate and minor levels, respectively
Reheat valve stuck	0%, 20%, 50%, 80%, and 100%	Assign a fixed simulated controlled device position
VAV damper stuck		
Reheat valve leaking	20%, 50%, and 80% of the max flow	Assign a 20%, 50%, and 80% of the max flow, respectively, when the valve is fully closed
Room air temperature control sequence unstable (PI1)	NA	Increase the proportional gain from 100 to 0.1 (for the PFPU) and 30 to 0.3 (for the SFPU) until the control is unstable
Room VAV damper control (PI2)	NA	Increase the proportional gain from 9.99 to 99999 until the control is unstable

**Table 7:** Methods of fault imposition for the chiller plant dataset.

Input Scenarios		Method of Fault Imposition
Fault Type	Fault Intensity	
Chiller 1: the chilled water leaving temperature sensor bias	−2 °C, −1 °C, 1 °C, and 2 °C	Add a bias to the sensor output
Cooling tower 1: the condenser water leaving temperature sensor bias		
In the secondary chilled water loop: the differential pressure sensor bias	−20%, −10%, 10%, and 20%	
The condenser water leaving the three-way valve leakage	25%, 50%, and 75%	Increase the default minimum position setting
The condenser water leaving the three-way valve stuck	50% and 75%	Assign a fixed device position
Cooling tower 1: heat exchanger fouling	95%, 80%, and 65%	Multiply intensity value by heat transfer coefficient
Controller PI for condenser loop supply temperature setpoint inappropriate tuning	NA	Modify the gain value of controllers

**Table 8:** Methods of fault imposition for the boiler plant dataset.

Input Scenarios		Method of Fault Imposition
Fault Type	Fault Intensity	
In the boiler 1, the hot water leaving temperature sensor bias	−2 °C, −1 °C, 1 °C, and 2 °C	Add a bias to the sensor output
In the hot water loop, the hot water leaving temperature sensor bias		
In the hot water loop, the differential pressure sensor bias	−20%, −10%, 10%, and 20%	
In the boiler 1, the heat exchanger fouling	65%, 80%, and 95%,	Multiply the intensity by the heat transfer coefficient
Controller PI for boiler supply temperature setpoint is inappropriate tuning	NA	Modify the gain value of controllers

**Table 9:** Methods of fault imposition for the experimental RTU dataset.

Input Scenarios		Method of Fault Imposition**
Fault Type	Fault Intensity	
OA damper stuck	Half of minimum position (5%), Minimum position (10%), 50% open, and fully open (100%)	Stuck the OA damper position at 5%, 10%, 50% and 100%; and stuck the RA damper position at 95%, 90%; 50% and 0%
Incorrect economizer setpoint*	6 °C, 8 °C, 12 °C and 12 °C	Modify the economizer setpoint in control programming
Biased supply air temperature sensor	2 °C, 4 °C, −2 °C and −4 °C	Modify the supply air temperature setpoint in control programming

*The correct economizer setpoint is 10 °C (50 °F)

**Faults were imposed in Fall 2020, Spring 2021, Summer 2021, and Winter 2022

**Table 10:** Methods of fault imposition for the simulated RTU dataset.

Input Scenario		Method of Fault Imposition
Fault Type	Fault Intensity	
Refrigerant overcharging	10%, 15%, and 20%	Lower refrigerant enthalpy. Less enthalpy results in higher charge because liquid has lower enthalpy, and high density
Refrigerant undercharging		Increase refrigerant enthalpy. More enthalpy results in lower charge because liquid has higher enthalpy, and low density
Condenser fouling	Reduction of airflow through the condenser (10%, 20%, 30%, 40%, and 50%)	Reduce condenser fan mass flow rate
Evaporator fouling		
Refrigerant liquid-line restriction	Pressure drops due to liquid-line restriction: (1 bar, 4 bar, 8 bar, and 10 bar)	Increase the pressure drop just after the condenser outlet (cold liquid refrigerant)
Refrigerant suction-line restriction	Pressure drops due to suction-line restriction: (1 bar, 3 bar, 6 bar, and 9 bar)	Increase the pressure drop just after the evaporator outlet (hot gas refrigerant)

The RTU dataset that was acquired from field measurements reflected a naturally occurring compressor staging fault and a refrigerant undercharging fault.

### Method of Brick schema model development

**Fig. 5:**
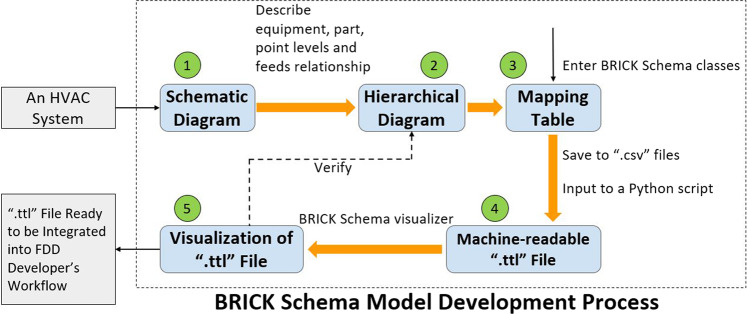
Flow of the Brick Schema model development.

The Brick schema^29^ offers classes and subclasses, of which the *equipment* class was used to designate the HVAC system components represented in the fault dataset. Similarly, the *point* subclass was used to design sensor measurement and control system data points. In addition, the schema offers ‘relationships’, of which *hasPart*, *hasPoint*, and *feeds*, are relevant to describing the fault dataset. Figure 5 illustrates the 5-step process that was used to generate the Brick models for each HVAC system in the dataset. Among them, Step 4 is automated while the other steps are performed manually.

### Step 1: Conceptualization of Brick relationships using mechanical drawing or schematic

The schematic representations for each system were reviewed to identify the major components for the overall system, to develop compositional (“hasPart”) relationships. For each major component, we identify all of the associated sensor/control points to develop “hasPoint” relationships. Lastly, we identify the order in which the given media (air, water, etc.) flow through the system to develop sequential (“feeds”) relationships between different equipment.

### Step 2: Creation of hierarchical diagram to visualize Brick relationships

After identifying the components and sensor/control points of the system in Step 1, we indicate which equipment has which components (“hasPart”), which equipment or component has which sensor and control data points (“hasPoint”), and which equipment feeds into another equipment (“feeds”).

### Step 3: Mapping system components to Brick classes

All equipment, components, and sensor/control data points in the hierarchical diagram are mapped to a Brick schema class and tabulated. The equipment and the sub components are mapped to a subclass of the Brick “equipment” class (e.g., chiller, AHU, and RTU) and the sensors and the control points will be assigned a type subclass of Brick “point”.

For each row (i.e., each component), we designate the relevant relationships, other components it connected to and these components. This way, we are able to incorporate all the components, their types and how they are related to other components.

### Step 4: Execution of script to create a .ttl file

The tables generated in Step 3 are exported as CSV files and imported to a Python script that generates a Brick model in the form of a machine-readable .ttl file. The script iterates through each row of the table, assigning all components and points to a specific instantiation of a Brick class and corresponding relationships. The .ttl file can be accessed by an FDD algorithm (or other applications), enabling more efficient and standardized retrieval of system metadata using SPARQL queries. This streamlines the interpretation of data semantics within the FDD or other applications.

### Step 5: Visualization of the Brick model to validate accuracy

The generated Brick model is verified by visualizing it and comparing it to the hierarchical diagram in Step 2. We used Brick Studio for the visualization and ensured that all the components in the data sets were present and the relationships between them were labeled correctly.

## Data Records

The data are stored on *figshare*^37^ and on an LBNL website^10^. The description for the expanded seven data sets can be found in Table 11. For each system, the FDD data are stored in individual comma separated value (CSV) files, and each file contains one fault type under one fault intensity. The data are stored at the 1-minute interval rate to reflect system operations. The 1-minute interval rate can be re-sampled to a 5-minute interval and a 15-minute interval, which are also commonly used in the existing building automation system (BAS). Time stamps are in the first column of each file, and presented in the format of “yyyymmdd hh:mm”.

**Table 11:** Files and size of each file in the full dataset, as well as system of focus and provenance.

System Type	System Description	Data Provenance	Number of Files	Total Data Size
Single-duct AHU	5 faults at various severity levels in a single-duct VAV AHU	Simulation using EnergyPlus-Modelica	23	593 MB
Chiller plant	7 faults at various severity levels in a chiller plant	Simulation using EnergyPlus-Modelica	22	1.14 GB
Boiler plant	5 faults at various severity levels in a boiler plant	Simulation using EnergyPlus-Modelica	17	330 MB
Rooftop unit	10 faults at various severity levels in 5 RTUs	Simulation using EnergyPlus-Modelica, experimental facilities, and field-measures	84*	293 MB
Dual-duct AHU	15 faults at various severity levels in a dual-duct VAV AHU	Simulation using HVACSIM+	58	1.65 GB
Fan-powered VAV terminal unit	10 faults at various severity levels in a parallel fan power unit and a series fan power unit, respectively	Simulation using HVACSIM+	66	2.04 GB
Fan coil unit	17 faults at various severity levels in a fan coil unit	Simulation using HVACSIM+	51	518 MB
RTU and AHU systems documented in Granderson *et al*.^9^	4–9 faults at various severity levels in single zone and multi-zone, constant and variable air volume AHUS, and in a rooftop unit	Simulation using HVACSIM+, EnergyPlus-Modelica, and an experimental facility	6	40.1 MB

*The RTU system includes three .ttl file and one inventory file.

Each system dataset is accompanied with a .ttl Brick model and also a data ‘inventory’ file that describes the key information necessary to understand the content and scope of each data set, including:

- An overview of the data set, who created it, and whether it was generated through simulation or physical experiment
- Building and system information
  - Model or experimental facility description
  - System type and physical configuration diagram
  - Control sequences
- Brick schema model diagram
- Data points
  - The unit for each measurement
  - The basic data points which existing BASs use are labeled
- Input scenarios for faulted and fault-free conditions represented in the data
  - Fault types
  - Fault intensities
  - Method of fault imposition

## Technical Validation

Granderson *et al*.^9^ documented that the validity of the dataset can be assessed according to three dimensions: (1) accuracy of the sensors and measurement infrastructure in the experimental facilities that were used; (2) accuracy of the simulation models that were used; and (3) accuracy of the ground truth labels that indicate the presence and severity of the faults, presence or absence of faults and their severity^9^.

### Facility measurement

Granderson *et al*. describes the measurement calibration process at the FLEXLAB, FRP, and Iowa Energy Center facilities^9^.

### Simulation models

Granderson *et al*. described the simulation model validation for the EnergyPlus-Modelica models^9^, and both Granderson *et al*. and Wen *et al*. describe model validation of the HVACSIM + models^9,35^. These publications describe a host of methods including empirical validation, experimental calibration, comparative testing (vs other tools), and analytical verification (with respect to exact solutions).

For the sake of brevity, the reader is referred to these prior publications for details on facility measurement and simulation model accuracy.

Granderson *et al*.^9^ describes a ground truth validation process that applies functional testing and engineering logic^9^. Functional testing verifies that system operation is consistent with the designed control sequences, and reflective of fault-free operational behavior. Engineering logic and the specified control sequence are combined to confirm that the data trends do indeed reflect the behaviors of the fault free and faulted scenarios.

**Fig. 6:**
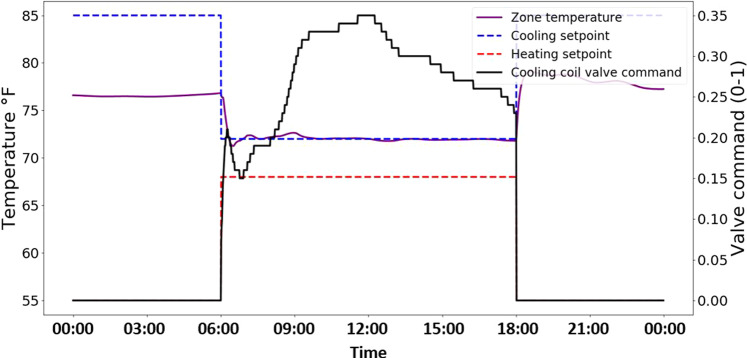
Example of FCU fault free operational data (July 17).

Figure 6 provides a few examples for the fan coil unit system. First the data trends are inspected to confirm that the system is operated according to the defined schedule of occupied hours corresponding to 6:00–17:59, and to the defined setpoints specified in the sequence (as shown in section *System configurations and control sequences*). This is verified in the profile of the cooling setpoint and heating setpoint trends, which respectively modulate from 85 °F to 72 °F, and from 55 °F to 68 °F, and back at the 6:00 and 17:59 timestamps. Next, the data trends are inspected to verify that the modeled PID parameters for the cooling valve controller are configured to output proper control signals. This is confirmed through smooth trend and absence of any significant oscillations in the plotted signal for the cooling coil valve command. Finally, inspection of the zone temperature trend confirms that the control objective, i.e., a cooling setpoint of 72 °F, was maintained throughout the occupied period of operation.

Following verification of the fault-free operational state, additional tests were conducted for each of the faulted scenarios. These tests considered (a) whether the imposed fault *condition* was correctly reflected in the data, and (b) whether the anticipated *symptoms* of the fault were reflected in other operational trends.

**Fig. 7:**
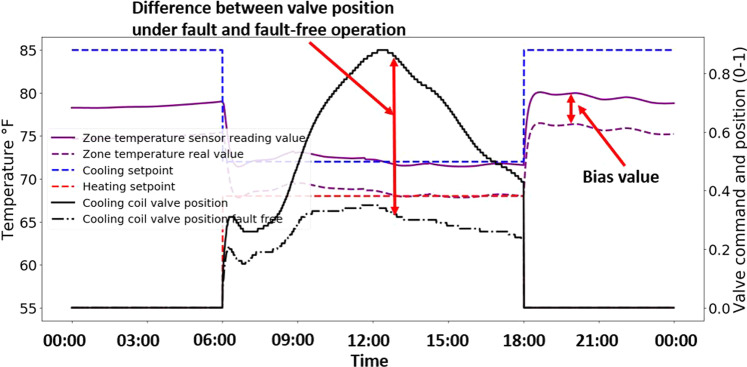
Example of FCU fault present operational data (zone air temperature sensor bias +2 °C (3.6 °F), July 17).

Figure 7 illustrates these two types of tests for the FCU system fault - zone air temperature sensor bias of +2 °C (3.6 °F). The biased condition is confirmed by comparing the 2 °C offset between the data trend from the ‘spoofed’ faulted model output point (solid line), and the unaltered output point (dashed line). This is clearly discernible and annotated in the righthand portion of the plot. The symptoms of this bias are observed in comparing the cooling coil valve position in the faulted case (the black solid line) to that from the unfaulted case (dashed black line). The position in the faulted case is significantly higher because the controller was attempting to provide an increased amount of cooling commensurate with the erroneously high zone air temperature reading.

**Fig. 8:**
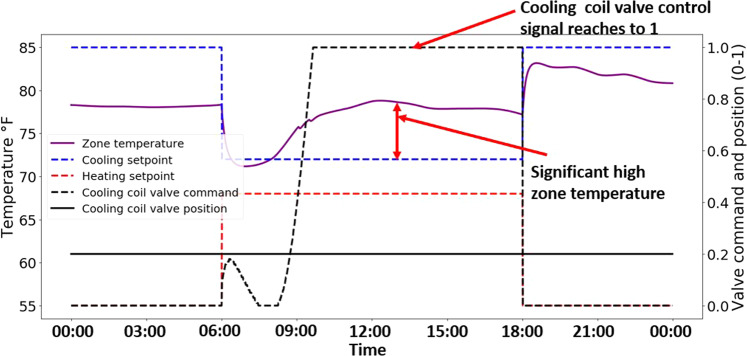
Example of FCU fault present operational data (cooling coil valve stuck fault at 20% position, July 17).

Figure 8 illustrates another FCU system fault - cooling coil valve stuck at 20%, imposed during the cooling season. Here, the faulted condition is confirmed by observing that the valve position signal (black solid line) is fixed at 0.2, while the valve command signal (black dashed line) is adjusted. The symptom of this fault is that the zone temperature (purple solid line) significantly exceeded the 72 °F cooling setpoint during the occupied hours even though the cooling coil valve control signal (black dashed line) reached a value of 1 (i.e., 100% position) in the controller’s attempt to provide maximum cooling.

Similar verification tests steps were performed for each fault type at each intensity level in experimental data sets and simulation data sets. For the simulated data sets that spanned a full year of operation, a sample of at least three days was selected for inspection from each of three operational seasons - summer/cooling season, winter/heating season and a transitional/swing season. This sampling enabled validation of the data and faulted system behaviors under different weather conditions and operational modes^38^.

## Usage Notes

A complete inventory of the data was developed to support users in interpreting the content and form of the data, and the corresponding HVAC systems, controls, and faults. The data itself comprise time series that can be analyzed with whatever software tools the user elects to implement. The data are provided at 1-minute intervals, and can be resampled as needed to fit the needs of specific applications.

## Data Availability

The Modelica Buildings Library and EnergyPlus are freely available for download^39^,^40^. EnergyPlus runs on Windows, Mac OSX, and Linux operating systems. A Windows or Linux-based computer and Dymola solver are required to run Modelica, and Dymola can be licensed from Modelica Buildings Library. HVACSIM + is also freely available, upon request from NIST, and has no operating system requirements. The Modelon air conditioning library that was used to model the faults in the RTU refrigerant side, was accessed from Modelon’s library suite^34^. The Modelica-based library is used to design, analyze and optimize air conditioning systems. A custom Python-based script was developed to create Brick model .ttl files for each system in the dataset, following the process described in *Method of Brick schema model development*. The .ttl files are included in the data repository.

## References

[1] Lin G, Kramer H, Nibler V, Crowe E, Granderson J (2022). Building Analytics Tool Deployment at Scale: Benefits, Costs, and Deployment Practices. Energies..

[2] Katipamula S, Brambley MR (2005). Methods for Fault Detection, Diagnostics, and Prognostics for Building Systems—A Review, Part I. HVAC&R Research..

[3] Katipamula S, Brambley MR (2005). Methods for Fault Detection, Diagnostics, and Prognostics for Building Systems—A Review, Part II. HVAC&R Research..

[4] Kim W, Katipamula S (2018). A review of fault detection and diagnostics methods for building systems. Science and Technology for the Built Environment..

[5] Li Y, O’Neill Z (2018). A critical review of fault modeling of HVAC systems in buildings. Build Simul..

[6] Pourarian S (2017). A tool for evaluating fault detection and diagnostic methods for fan coil units. Energy and Buildings..

[7] Zhang R, Hong T (2017). Modeling of HVAC operational faults in building performance simulation. Applied Energy..

[8] Comstock, M., Braun, J. *Development of Analysis Tools for the Evaluation of Fault Detection and Diagnostics in Chillers, ASHRAE Research Project RP-1043*. Ray W. Herrick Laboratory Purdue University; (1999).

[9] Granderson J, Lin G, Harding A, Im P, Chen Y (2020). Building fault detection data to aid diagnostic algorithm creation and performance testing. Sci Data..

[10] Granderson, J., Lin, G., Chen, Y., Casillas, A. *LBNL Fault Detection and Diagnostics Datasets*. DOE Open Energy Data Initiative (OEDI); Lawrence Berkeley National Lab. (LBNL), Berkeley, CA (United States); https://data.openei.org/submissions/5763, https://faultdetection.lbl.gov/, 10.25984/1881324 (2022).

[11] Comstock MC, Braun JE, Groll EA (2002). A survey of common faults for chillers. ASHRAE Transactions..

[12] Crowe, E. *et al*. What We Learned From Analyzing 15 Million Rows of Commercial Buildings’ HVAC Fault Data. In: *2022 Summer Study on Energy Efficiency in Buildings Proceedings*. (2022).

[13] Kim J (2021). Research challenges and directions in HVAC fault prevalence. Science and Technology for the Built Environment..

[14] Chen Y, Lin G, Chen Z, Wen J, Granderson J (2022). A simulation-based evaluation of fan coil unit fault effects. Energy and Buildings..

[15] Li Y, O’Neill Z (2019). An innovative fault impact analysis framework for enhancing building operations. Energy and Buildings..

[16] Roth, K., Westphalen, D., Llana, P., Feng, M. The Energy Impact of Faults in U.S. Commercial Buildings. In: *International Refrigeration and Air Conditioning Conference*. Purdue University; 2004.

[17] Wang S, Xiao F (2004). AHU sensor fault diagnosis using principal component analysis method. Energy and Buildings..

[18] Breuker MS, Braun JE (1998). Common faults and their impacts for rooftop air conditioners. HVAC&R Research..

[19] Salah M, Osman H, Hosny O (2018). Performance-Based Reliability-Centered Maintenance Planning for Hospital Facilities. Journal of Performance of Constructed Facilities..

[20] Gunay B (2018). Energy and comfort performance benefits of early detection of building sensor and actuator faults. Building Services Engineering Research and Technology..

[21] Chen, Y., Chen, Z., Lin, G., Wen, J., Granderson, J. A Simulation-Based Method to Analyze Fan Coil Unit Fault Impacts. In: *ASHRAE Transactions*. **Vol 128**. ASHRAE; 210. https://www.ashrae.org/technical-resources/ashrae-transactions (2023).

[22] Ginestet S, Marchio D, Morisot O (2008). Evaluation of faults impacts on energy consumption and indoor air quality on an air handling unit. Energy and Buildings..

[23] Granderson, J., G.R. *et al*. *Commercial Fault Detection and Diagnostics Tools: What They Offer, How They Differ, and What’s Still Needed*. Lawrence Berkeley National Lab.(LBNL), Berkeley, CA (United States); 2018. https://escholarship.org/uc/item/4j72k57p Accessed November 23, (2021).

[24] Frank S (2019). A performance evaluation framework for building fault detection and diagnosis algorithms. Energy and Buildings..

[25] Yuill DP, Braun JE (2013). Evaluating the performance of fault detection and diagnostics protocols applied to air-cooled unitary air-conditioning equipment. HVAC&R Research..

[26] Lin G, Pritoni M, Chen Y, Granderson J (2020). Development and Implementation of Fault-Correction Algorithms in Fault Detection and Diagnostics Tools. Energies..

[27] Pritoni M (2022). From fault-detection to automated fault correction: A field study. Building and Environment..

[28] Eskandari N, Noorzai E (2021). Offering a preventive solution to defects in commercial building facility system using BIM. Facilities..

[29] Balaji B (2018). Brick: Metadata schema for portable smart building applications. Applied Energy..

[30] Clark, D. R., May W. B. *HVACSIM+ Building Systems and Equipment Simulation Program - User’s Guide*. National Bureau of Standards, Building Equipment Division; 1985. https://www.osti.gov/biblio/6127383 Accessed November 20, (2021).

[31] Wetter M, Zuo W, Nouidui TS, Pang X (2014). Modelica Buildings library. Journal of Building Performance Simulation..

[32] Crawley DB (2001). EnergyPlus: creating a new-generation building energy simulation program. Energy and Buildings..

[33] Pourarian S, Kearsley A, Wen J, Pertzborn A (2016). Efficient and robust optimization for building energy simulation. Energy and Buildings..

[34] Modelon. Air Conditioning Library. Modelon. Published 2020. https://modelon.com/library/air-conditioning-library/Accessed October 31, (2022).

[35] Wen, J., Pourarian, S., Yang, X., Li, X. *NIST 10D243 Tools for Evaluating Fault Detection and Diagnostic Methods for HVAC Secondary Systems of a Net Zero Building*. The US National Institute of Standard & Technology; 2015. https://www.researchgate.net/profile/Jin-Wen-12/publication/337946061_NIST10D243_FinalReport_-Tools_for_Evaluating_Fault_Detection_and_Diagnostic_Methods_for_HVAC_Secondary_Systems_of_a_Net_Zero_Building/links/5df73f11299bf10bc35f1163/NIST10D243-FinalReport-Tools-for-Evaluating-Fault-Detection-and-Diagnostic-Methods-for-HVAC-Secondary-Systems-of-a-Net-Zero-Building.pdf Accessed January 25, (2021).

[36] Wheeler, G., Kozubal, E., Judkoff, R. Experimental Design and Laboratory Characterization of a Medium- and High-Efficiency Rooftop Unit for use in Building Energy Simulations. *International Refrigeration and Air Conditioning Conference*. https://docs.lib.purdue.edu/iracc/2057 Published online January 1, (2018).

[37] Granderson J (2023). figshare.

[38] Casillas, A., Lin, G., Granderson, J. Curation of Ground-Truth Validated Benchmarking Datasets for Fault Detection Diagnostics Tools. In: *2020 ACEEE Summer Study on Energy Efficiency in Buildings*.; 2020. https://escholarship.org/uc/item/9cz491j6 Accessed November 20, (2021).

[39] Lawrence Berkeley National Lab. Modelica Buildings Library. Published December 6, **2022**. https://simulationresearch.lbl.gov/modelica.html Accessed January 1, (2023).

[40] Department of Energy, U.S. EnergyPlus. Published 2022. https://energyplus.net/ Accessed January 1 (2023).

